# Using threshold Cox models to estimate change points in exposure-response relationships in an occupational epidemiological study of respirable crystalline silica and silicosis risk

**DOI:** 10.3389/fpubh.2025.1628965

**Published:** 2025-09-19

**Authors:** Diezhang Wu, Kenneth A. Mundt, Jing Qian

**Affiliations:** Department of Biostatistics and Epidemiology, University of Massachusetts Amherst, Amherst, MA, United States

**Keywords:** change point, exposure-response relationship, Firth's penalized likelihood, heavy censoring, occupational epidemiology, respirable crystalline silica, silicosis, threshold Cox model

## Abstract

**Introduction:**

In occupational epidemiology, accurately quantifying exposure-response relationships is crucial.

**Methods:**

We introduce a threshold Cox model that includes a change point term to identify the optimal threshold. To address bias associated with maximum likelihood estimation under monotone likelihood, we employ Firth's penalized likelihood approach. The methodology was validated using simulation studies that evaluated model performance under various censoring rates and sample sizes. We applied our threshold Cox model to data from an occupational epidemiological study of respirable crystalline silica (RCS) exposure and risk of silicosis (defined as ILO category 1/0 or higher). To improve the condition of the data for analysis using Cox regression, which is sensitive to small proportions of events, we included all silicosis cases, and for each case we density-sampled four non-cases from workers in the same production areas (mostly materials preparation).

**Results:**

Thresholds for (a) cumulative RCS exposure and (b) average RCS exposure intensity over 2 years and 5 years were identified as 4.038 mg/m^3^-years (95 CI: 3.109–4.967), and 0.264 mg/m^3^ (95 CI: 0.207–0.321), and 0.324 mg/m^3^ (95 CI: 0.263–0.385), respectively.

**Results and Discussion:**

These quantified exposure thresholds may be useful in verifying that occupational exposure limits are protective against silicosis and for quantitative risk assessment. This methodology also could be applied to other exposure-disease relationships to identify and quantify possible exposure thresholds.

## 1 Introduction

The Cox model is frequently used in epidemiological studies to examine the association between environmental exposure levels and health outcome. Typically, continuous exposure levels are categorized, and the results help determine whether certain exposure levels carry a higher risk compared to a reference group. For instance, a study by Birk et al. (1) explored the risk of silicosis associated with quantitative estimates of occupational exposure to respirable crystalline silica (RCS). They reported increased risk among workers in the categories with estimated average exposure > 0.15 mg/m^3^ and cumulative exposure > 1.0 mg/m^3^-years, respectively. However, conventional Cox models with transformed exposure variables fail to provide precise estimates of the exposure level above which risk is statistically significantly increased above background, especially when the incremental exposure categories analyzed are wide. This undefined value is referred to as an exposure threshold or change point. In this paper, we introduce a threshold Cox model capable of simultaneously identifying optimal threshold values and the corresponding regression coefficients and 95% confidence intervals. We also extend this model to accommodate situations where outcomes are relatively rare, and illustrate the methods using the data from Birk et al. (1), in which silicosis was diagnosed in < 1% of the total cohort.

Following this introduction, we provide details on the notation of the proposed model in Section 2, along with two estimation procedures to obtain parameter estimates. In Section 3, we present two simulation studies to validate the model and evaluate its performance under different scenarios. We then apply the model to real-world data from a recently published occupational epidemiological study in Section 4. Lastly, in Section 5 we briefly discuss the key findings, strengths and weaknesses, and provide ideas for future research.

## 2 Methodology

### 2.1 Threshold Cox model with change point

#### 2.1.1 Notations of the threshold Cox model

Consider a cohort of *n* independent individuals, where each individual *i* is associated with an exposure level *Z*_*i*_, such as the concentration of respiratory silica in the air, and a vector of covariates $X_{i}={\left(X_{i1},X_{i2},\ldots ,X_{ip}\right)}^{\text{T}}$, which might include age, gender and smoking status. The time to the event of interest, such as the onset of a disease, is denoted by *T*_*i*_. The time at which the individual is right-censored (i.e., the event has not occurred by the end of follow-up) is presented by *C*_*i*_. The observed data consist of the observed event time *Y*_*i*_ = min(*T*_*i*_, *C*_*i*_), and the censoring indicator δ_*i*_ = *I*(*T*_*i*_ ≤ *C*_*i*_), which equals 1 if the event is observed and 0 if the observation is right-censored. In a standard Cox model (2), the hazard function λ(*t*) at time *t* is modeled as a product of the baseline hazard λ_0_(*t*) and an exponential function of a linear combination of covariates and exposure levels:


(1) $$\begin{array}{l} \lambda \left(t\right)=\lambda_{0}\left(t\right)\exp\left\{\beta^{\text{T}}X+\alpha Z\right\}. \end{array}$$

Here, $\beta ={\left(\beta_{1},\beta_{2},\ldots ,\beta_{p}\right)}^{\text{T}}$ are the coefficients associated with the covariates ***X***, and α is the coefficient representing the effect of the exposure *Z* on the hazard function. However, this model assumes that the effect of the exposure remains constant across all levels of *Z*, which may be unrealistic in cases where the exposure increases the hazard rate only above a certain exposure threshold, or when there is a shift in the effect of exposure at a certain point (after which the risk plateaus, e.g., a step function).

To account for this possibility, the threshold Cox model introduces a change point τ, representing a certain exposure level at which the effect of *Z* on the hazard function changes. The threshold Cox model is expressed as:


(2) $$\begin{array}{l} \lambda \left(t\right)=\lambda_{0}\left(t\right)\exp\left\{\beta^{\text{T}}X+\alpha Z+\gamma {\left(Z-\tau \right)}^{+}\right\}, \end{array}$$

where (*Z*−τ)^+^ = max(0, *Z*−τ). This formula captures the idea that the effect of the exposure *Z* on the log hazard ratio changes at rate α for values below the threshold τ, but once the exposure exceeds τ, an additional effect γ is introduced. The threshold Cox model is highly versatile, as it can be further extended to include multiple change points or to accommodate more complex interactions between covariates and exposures. These extension, however, are beyond the scope of the present work.

#### 2.1.2 Partial likelihood function of the threshold Cox model

In a standard Cox model (Equation 1), regression coefficients are estimated through maximizing the partial likelihood function (3), which is given by:


(3) $$\begin{array}{l} L\left(\beta^{\text{T}},\alpha \right)=\overset{n}{\underset{i=1}{\prod }}{\left[\frac{\exp\left\{\beta^{\text{T}}X_{i}+\alpha Z_{i}\right\}}{\overset{n}{\underset{k=1}{\sum }}I\left(Y_{k}\geq Y_{i}\right)\exp\left\{\beta^{\text{T}}X_{k}+\alpha Z_{k}\right\}}\right]}^{\delta_{i}}. \end{array}$$

The exponentiation by δ_*i*_ ensures that only the terms corresponding to uncensored events contribute to the product in the likelihood function. The contribution of censored observations is indirectly handled through the risk set $\overset{n}{\underset{k=1}{\sum }}I\left(Y_{k}\geq Y_{i}\right)\exp\left\{\beta^{\text{T}}X_{k}+\alpha Z_{k}\right\}$, which represents the set of individuals still at risk of experiencing the event of interest at the time *Y*_*i*_, when the event occurs for the *i*-th individual. The partial likelihood circumvents the need to specify the baseline hazard function parametrically, rendering the Cox model a flexible semi-parametric approach.

The partial likelihood function of the threshold Cox model (Equation 2) is similar to that of the standard Cox model, as shown in Equation 3, but includes additional complexity due to the presence of the change point. The partial likelihood function for the threshold Cox model is given by:

(4) $$L(\beta^{\text{T}},\alpha ,\gamma ,\tau )=\prod_{i=1}^{n}{\left[\frac{\exp\{\beta^{\text{T}}X_{i}+\alpha Z_{i}+\gamma {\left(Z_{i}-\tau \right)}^{+}\}}{\begin{array}{c} \sum_{k=1}^{n}I(Y_{k}\geq Y_{i})\exp\{\beta^{\text{T}}X_{k}+\alpha Z_{k}\\ \;+\gamma {\left(Z_{k}-\tau \right)}^{+}\} \end{array}}\right]}^{\delta_{i}}.$$

In addition to regression coefficients ***β*** and α, the threshold Cox model simultaneously estimates the threshold effect coefficient γ and the change point τ.

### 2.2 Estimation procedure

#### 2.2.1 Two-step grid search procedure

To estimate (***β***^T^, α, γ, τ)T in the threshold Cox model, we developed a two-step grid search procedure, which explores candidate values of τ within a pre-specified range to identify the optimal change point where the partial likelihood is maximized. In this procedure, we first construct a grid over the interval [*a, b*], which is assumed to contain the true value of the change point τ, with a chosen resolution. For each candidate value ξ on the grid, we compute the maximum partial likelihood with respect to (***β***^T^, α, γ)T. We then select the value of ξ that yields the overall maximum of these maximized partial likelihoods as the estimated change point $\hat{\tau }$. The corresponding parameter estimates ${\left({\hat{\beta }}^{\text{T}},\hat{\alpha },\hat{\gamma }\right)}^{\text{T}}$ are maximum likelihood estimates (MLE) obtained from the partial likelihood maximized at $\hat{\tau }$. The following procedure outlines the algorithm step-by-step.

Procedure 1. Two-step grid search method.

**Step 1: Finding optimal change point**$\hat{\tau }$ **1.1:** Define the grid range. Create a grid $\xi^{\text{T}}=\left\{\xi_{1},\xi_{2},\ldots ,\xi_{M}\right\}$, where *a* = min(*Z*_1_, …, *Z*_*n*_) ≤ ξ_1_ < ⋯ < ξ_*M*_ ≤ max(*Z*_1_, …, *Z*_*n*_) = *b*, and ξ_*m*_−ξ_*m*−1_ = ε for *m* = 2, …, *M*. Here, the interval [*a, b*] contains the true change point value τ^*^, and ε is the resolution of this grid.**1.2:** Calculate the maximum partial likelihood for each grid point. For each possible value ξ_*m*_ in the grid, maximize the partial likelihood with respect to (***β***^T^, α, γ)T and evaluate the partial likelihood function, as defined in Equation 4, at the corresponding maximum likelihood estimates, i.e., $L\left({\hat{\beta }}_{m}^{\text{T}},{\hat{\alpha }}_{m},{\hat{\gamma }}_{m},\xi_{m}\right)$.**1.3:** Select the optimal change point. Once the maximum partial likelihood has been computed for all ξ's, the optimal change point $\hat{\tau }$ is defined as the value of ξ that yields the overall maximum of these maximized partial likelihoods:

$$\hat{\tau }=\underset{\xi_{m}\in \xi }{\mathrm{argmax}}L\left({\hat{\beta }}_{m}^{\text{T}},{\hat{\alpha }}_{m},{\hat{\gamma }}_{m},\xi_{m}\right).$$

**Step 2: Estimating model parameters**. ${\left({\hat{\beta }}^{\text{T}},\hat{\alpha },\hat{\gamma }\right)}^{\text{T}}$. Obtaining the corresponding parameter estimates ${\left({\hat{\beta }}^{\text{T}},\hat{\alpha },\hat{\gamma }\right)}^{\text{T}}$ from the partial likelihood maximized at $\hat{\tau }$.

However, since the change point $\hat{\tau }$ is selected from a pre-specified grid, the algorithm does not directly provide a variance estimate for $\hat{\tau }$ or account for its associated uncertainty. Instead, the variance of $\hat{\tau }$ can be estimated using a non-parametric bootstrap method.

The two-step grid search method is intuitive, computationally feasible, and provides robust estimates of the model parameters. It simplifies the optimization process by focusing on discrete values of the change point, making it more manageable compared to continuous optimization methods. Additionally, this method is flexible and can be adapted to different resolutions and ranges of the grid, allowing for fine-tuning based on the data and computational resources available.

While the method efficiently estimates the change point and the corresponding regression coefficients, the use of bootstrap resampling to estimate the variance can be computationally intensive, particularly for large sample sizes. Moreover, the estimation of $\hat{\tau }$ depends on the range and resolution of the grid, which are selected arbitrarily and may lead to inaccurate results if not chosen appropriately.

#### 2.2.2 One-step MLE via maxLik()

To address the previously mentioned drawbacks of the two-step grid search method, we proposed a one-step MLE approach, implemented using the R function maxLik() in the maxLik R package. This method offers unbiased estimation for the change point and regression coefficients. Unlike the grid-search method, which estimates parameters in stages, the one-step MLE provides simultaneous estimation of both the parameters and their corresponding analytical variances.

The maxLik() function in R optimizes the likelihood function through a Newton-Raphson algorithm, which iteratively updates parameter estimates to maximize the log-likelihood. To perform MLE estimation of the threshold Cox model using maxLik(), three key inputs are required:

- **Log-likelihood function**: the log-likelihood function of the threshold Cox model is given by:
  (5) $$\begin{array}{l} \log\left[L(\beta^{\text{T}},\alpha ,\gamma ,\tau )\right]=\sum_{i=1}^{n}\{\left[\beta^{\text{T}}X_{i}+\alpha Z_{i}+\gamma {\left(Z_{i}-\tau \right)}^{+}\right]\\ \;-\log[\sum_{k=1}^{n}I(Y_{k}\geq Y_{i})\exp\{\beta^{\text{T}}X_{k}\\ \;+\alpha Z_{k}+\gamma {\left(Z_{k}-\tau \right)}^{+}\}]\}\delta_{i}. \end{array}$$
- **Analytical gradient function**: the gradient function contains the first-order partial derivatives of the log-likelihood Equation 5 with respect to (***β***^T^, α, γ, τ)T, which are given by
  $$\begin{array}{l} \frac{\partial \log L}{\partial \beta^{\text{T}}}=\sum_{i=1}^{n}\left[X_{i}-\frac{\begin{array}{c} \sum_{k=1}^{n}I(Y_{k}\geq Y_{i})X_{k}\exp\{\beta^{\text{T}}X_{k}\\ +\alpha Z_{k}+\gamma {\left(Z_{k}-\tau \right)}^{+}\} \end{array}}{\begin{array}{c} \sum_{k=1}^{n}I(Y_{k}\geq Y_{i})\exp\{\beta^{\text{T}}X_{k}\\ +\alpha Z_{k}+\gamma {\left(Z_{k}-\tau \right)}^{+}\} \end{array}}\right]\delta_{i},\\ \frac{\partial \log L}{\partial \alpha }=\sum_{i=1}^{n}\left[Z_{i}-\frac{\begin{array}{c} \sum_{k=1}^{n}I(Y_{k}\geq Y_{i})Z_{k}\exp\{\beta^{\text{T}}X_{k}\\ +\alpha Z_{k}+\gamma {\left(Z_{k}-\tau \right)}^{+}\} \end{array}}{\begin{array}{c} \sum_{k=1}^{n}I(Y_{k}\geq Y_{i})\exp\{\beta^{\text{T}}X_{k}\\ +\alpha Z_{k}+\gamma {\left(Z_{k}-\tau \right)}^{+}\} \end{array}}\right]\delta_{i},\\ \frac{\partial \log L}{\partial \gamma }=\sum_{i=1}^{n}[{\left(Z_{i}-\tau \right)}^{+}\\ \;-\frac{\begin{array}{c} \sum_{k=1}^{n}I(Y_{k}\geq Y_{i}){\left(Z_{k}-\tau \right)}^{+}\exp\{\beta^{\text{T}}X_{k}\\ +\alpha Z_{k}+\gamma {\left(Z_{k}-\tau \right)}^{+}\} \end{array}}{\begin{array}{c} \sum_{k=1}^{n}I(Y_{k}\geq Y_{i})\exp\{\beta^{\text{T}}X_{k}\\ +\alpha Z_{k}+\gamma {\left(Z_{k}-\tau \right)}^{+}\} \end{array}}]\delta_{i}, \end{array}$$
  and
  $$\begin{array}{l} \frac{\partial \log L}{\partial \tau }=\sum_{i=1}^{n}[(-\gamma )I(Z_{i}>\tau )\;\\ \;-\frac{\begin{array}{c} \sum_{k=1}^{n}I(Y_{k}\geq Y_{i})(-\gamma )I(Z_{k}>\tau )\\ \exp\{\beta^{\text{T}}X_{k}+\alpha Z_{k}+\gamma {\left(Z_{k}-\tau \right)}^{+}\} \end{array}}{\begin{array}{c} \sum_{k=1}^{n}I(Y_{k}\geq Y_{i})\exp\{\beta^{\text{T}}X_{k}+\alpha Z_{k}\\ +\gamma {\left(Z_{k}-\tau \right)}^{+}\} \end{array}}]\delta_{i}. \end{array}$$
  These four terms indicate the direction in which the likelihood increases most rapidly. The Newton–Raphson algorithm uses the gradient to iteratively refine parameter estimates until convergence.
- **Initial parameter values**: the optimization process requires specifying initial values for the parameter vector (***β***^T^, α, γ, τ)T to initialize the iterative estimation process. An initial value for τ may be chosen within its allowable range based on subject-matter knowledge. Conditional on this value of τ, the maximum likelihood estimates of (***β***^T^, α, γ)T can be obtained following Step 1.2 in the two-step grid search procedure. These estimates are then used as the initial values for (***β***^T^, α, γ)T.

This one-step MLE approach overcomes the limitations of the grid search method by providing unbiased point estimates and the variance associated with the point estimates in a single, streamlined process. It offers a comprehensive solution for fitting the threshold Cox model to survival data with a change point. While some may prefer bootstrap methods for variance estimation due to their flexibility and robustness, our approach offers a direct and efficient solution for fitting the threshold Cox model to survival data with change points. We will further discuss the relative merits of bootstrap-based inference in Section 4.

### 2.3 Firth's penalized MLE for monotone likelihood under extreme censoring

In survival analysis, particularly under certain challenging conditions, including extreme censoring or the presence of strong covariates, the MLE approach may result in biased estimates. This phenomenon, referred to as monotone likelihood, occurs due to the non-existence of a true maximum likelihood in such cases (4, 5). When modeling datasets with monotone likelihood, convergence issues often arise, leading to severely biased parameter estimates. This issue needed to be addressed, as the example in which we apply the threshold Cox model reflects a real-world scenario where the silicosis outcome (i.e., failure) is rare and the censoring rate in our dataset exceeds 99%. Such extreme censoring rates introduce monotone likelihood, and require adjustment to prevent the bias.

Firth's penalization method, which has been widely recommended in the literature (6–8), is used to adjust the MLE of the threshold Cox model to reduce biases caused by monotone likelihood. Firth's approach modifies the estimation process by applying a small penalty term to the likelihood function. Let *L* denote the partial likelihood function and I the information matrix for a standard Cox model, the penalized likelihood function proposed by Firth is given by:


(6) $$\begin{array}{l} \log L_{Firth}=\log L+0.5\log|I|. \end{array}$$

Here, the penalty term 0.5log|I|, also known as Jeffrey's invariant prior, is asymptotically negligible. It prevents the likelihood function from becoming infinite and ensures that the MLE converges to a reasonable value.

The penalized likelihood function (Equation 6) can be extended to the threshold Cox model. In this case, the penalized log partial likelihood function takes the form:


(7) $$\begin{array}{lll} \log\left[L_{Firth}\left(\beta^{\text{T}},\alpha ,\gamma ,\tau \right)\right] & = & \log L\left(\beta^{\text{T}},\alpha ,\gamma ,\tau \right)\\ \;+ & 0.5\log|I\left(\beta^{\text{T}},\alpha ,\gamma ,\tau \right)|, \end{array}$$

where log*L*(***β***^T^, α, γ, τ) is defined in Equation 5, $\left|I\left(\beta^{\text{T}},\alpha ,\gamma ,\tau \right)\right|$ is the observed information matrix that is defined as:


$$\begin{array}{l} I\left(\beta^{\text{T}},\alpha ,\gamma ,\tau \right)=-E\left[\frac{\partial^{2}\log L\left(\beta^{\text{T}},\alpha ,\gamma ,\tau \right)}{\partial \left(\beta^{\text{T}},\alpha ,\gamma ,\tau \right)\partial {\left(\beta^{\text{T}},\alpha ,\gamma ,\tau \right)}^{\text{T}}}\right]. \end{array}$$

To optimize the penalized log-likelihood function (Equation 7) with maxlik(), we employ the score function, which incorporates the derivative of the penalty term using Jacobi's formula. The score function of the penalized log-likelihood is given by:


$$\begin{array}{l} \frac{\partial \log L_{Firth}\left(\beta^{\text{T}},\alpha ,\gamma ,\tau \right)}{\partial \left(\beta^{\text{T}},\alpha ,\gamma ,\tau \right)}=\frac{\partial \log L\left(\beta^{\text{T}},\alpha ,\gamma ,\tau \right)}{\partial \left(\beta^{\text{T}},\alpha ,\gamma ,\tau \right)}\\ \;+\frac{1}{2}\text{tr}[ℐ{\left(\beta^{\text{T}},\alpha ,\gamma ,\tau \right)}^{-1}\\ \;\cdot \frac{\partial ℐ\left(\beta^{\text{T}},\alpha ,\gamma ,\tau \right)}{\partial \left(\beta^{\text{T}},\alpha ,\gamma ,\tau \right)}], \end{array}$$

where tr(**A**) denotes the trace of a square matrix **A**, i.e., the sum of the elements on its main diagonal.

### 2.4 Software

All statistical analyses were conducted using R version 4.4.0 (9). The MLE estimation was performed with R package “maxLik” version 1.5-2.1 (10). A sample code showing the application of maxLik() with a simulated dataset is attached in the Supplementary material.

## 3 Simulation study

### 3.1 Simulation set up

We conducted two simulation studies under varying conditions to evaluate the robustness and performance of the proposed estimation procedures for the threshold Cox model. The datasets for both simulation studies were constructed from the same data generation step. We first generated the covariate *X* and the exposure *Z*, where *X* follows a Bernoulli distribution with Pr(*X* = 1) = 0.55 and *Z* follows an exponential distribution with rate parameter 0.5. The survival time *T* was then simulated based on a simple version of the proposed threshold Cox model, $\lambda \left(t\right)=\lambda_{0}\left(t\right)\exp\left\{\beta^{*}X+\alpha^{*}Z+\gamma^{*}{\left(Z-\tau^{*}\right)}^{+}\right\}$, using true parameter values (β^*^, α^*^, γ^*^, τ^*^) = (0.75, 0.25, 0.5, 4). The censoring time *C* is drawn from a uniform distribution *U*(*a, b*), with different combinations of *a* and *b* to control the censoring rate. The observed time *Y* = min(*T, C*) and the censoring indicator δ = *I*(*T* ≤ *C*) are derived from the simulated event time and censoring time. We ran 1,000 simulations for each scenario. The evaluation metric for both studies includes bias, mean squared error (MSE), and coverage probability, which helps to interpret the results and provides insight into the model's reliability under different data constraints.

### 3.2 Study 1: model validation under various censoring rates

The first simulation study aims to validate the accuracy and stability of the model across different censoring rates. For each replication, we generated five simulated datasets of size *N*= 10,000 observations. These datasets were simulated for increasing rates of censoring at 20%, 40%, 60%, 85%, and 98%. The dataset with a censoring rate of 98% was intended to demonstrate the situation where monotone likelihood occurs. R package segmented() can be used to fit regression models with broken-line relationship for survival outcomes (11), similar to the approach described in this paper. We included coefficient estimation using segmented() in our simulation for comparison.

**Figure 1:**
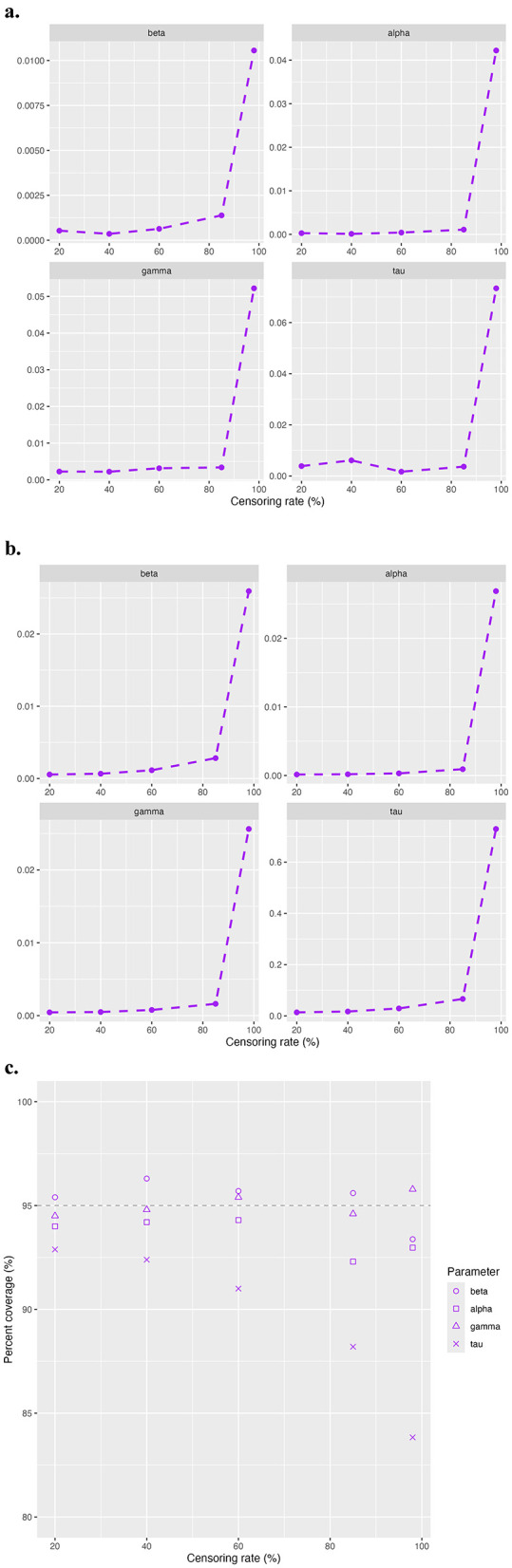
Three panels displaying statistical data against censoring rates. Panel (a) shows absolute bias for parameters beta, alpha, gamma, and tau, each with a steep increase at higher censoring rates. Panel (b) illustrates mean squared error (MSE) for the same parameters, also rising sharply with higher rates. Panel (c) presents 95% confidence interval (CI) coverage across censoring rates for the parameters, shown via scattered points. Each panel demonstrates the impact of censoring on bias, error, and coverage. Simulation results for study 1 model validation. The evaluation metric includes **(a)** absolute bias, **(b)** MSE, and **(c)** coverage rates of 95% CI.

**Figure 2:**
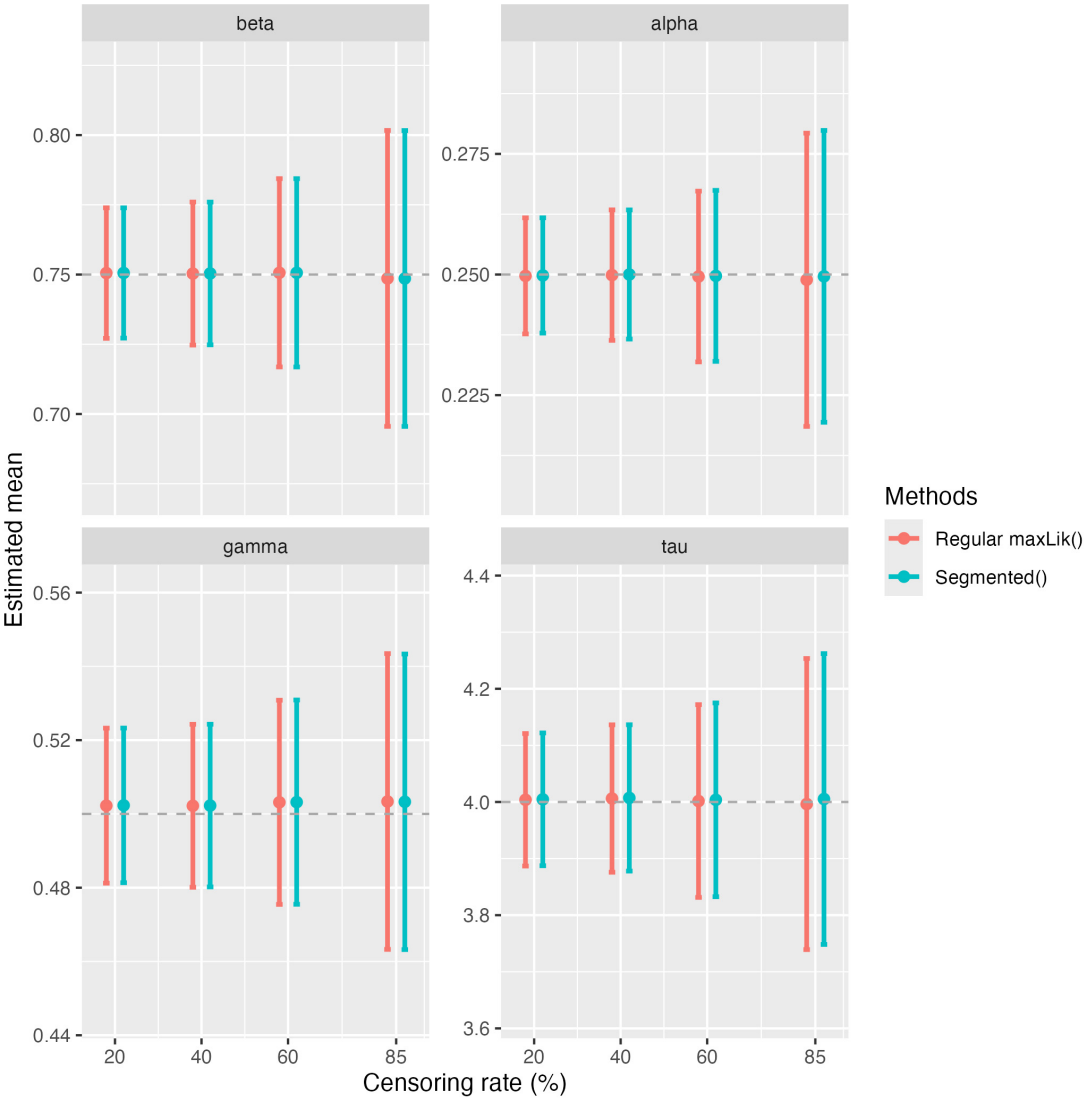
Comprison between parameter estimations using maxLik() and Segmented().

We present in Figure 1 the simulation results in terms of absolute bias (Figure 1a), MSE (Figure 1b), and empirical coverage rates of 95% confidence interval (CI) (Figure 1c) for β, α, γ, and τ. For all parameters, the absolute biases remain close to zero at the first four censoring rates of 20%, 40%, 60% and 85%, but spike at the extreme censoring rate of 98%. A similar pattern is observed for the MSE, which increases slightly with rising censoring rates before increasing drastically at 98%. Coverage rates for the 95% CI of β, α, and γ remain close to the nominal 95% threshold for all levels of censoring. However, for τ, the coverage rate decreases as the censoring rate increases. Figure 2 provides a visual comparison of point estimates and 95% CIs obtained with the standard maxLik() and Segmented() approaches at non-extreme censoring rates. The results indicate that both methods yield highly comparable point estimates and uncertainty levels, suggesting similar performance in these scenarios.

Despite adding the penalty term, some replications at 98% censoring rate still fail to converge due to the high censoring rate. To ensure a fair comparison, we excluded the replications that failed to converge and summarized the simulation results for the regular, penalized, and segmented approaches in Table 1. As shown, the penalized model generally results in lower absolute bias compared to the regular model for β and τ, but higher bias for α and γ. For instance, $\hat{\beta }$ has a smaller absolute bias in the penalized model, whereas $\hat{\alpha }$ has a higher bias at 0.05 compared to 0.04. In addition, the penalized model generates lower or comparable MSE across all parameters, most notably for τ. All models produce similar analytical SE values across the parameters, with slightly lower SEs observed in the penalized model. The regular model tends to provide higher coverage rates than the penalized model, particularly for τ where the coverage drops from 85.1% in the regular model to 77.8% in the penalized model. In summary, the penalized model improves bias and MSE for certain parameters, particularly for β and τ, at the cost of slightly reduced coverage for some parameters.

**Table 1:** Absolute bias, MSE, analytical SE, and empirical coverage rate of 95% CI using the regular, penalized maxLik() and Segmented() approaches with adjusted number of replications under 98% censoring rate.

**Param**	**Abs. bias(% Rel. bias)**			**MSE**			**Analy SE**			**Coverage**		
	**Reg**.	**Pen**.	**Seg**.	**Reg**.	**Pen**.	**Seg**.	**Reg**.	**Pen**.	**Seg**.	**Reg**.	**Pen**.	**Seg**.
β	0.01 (1.6)	0.00 (0.5)	0.01 (1.6)	0.03	0.03	0.03	0.16	0.16	0.16	93.0	93.4	93.0
α	0.04 (15.2)	0.05 (20.4)	0.03 (12.9)	0.02	0.02	0.02	0.10	0.10	0.10	93.4	93.0	92.8
γ	0.05 (9.4)	0.07 (13.2)	0.04 (8.5)	0.02	0.02	0.02	0.12	0.11	0.12	96.3	93.8	96.7
τ	0.07 (1.7)	0.03 (0.7)	0.02 (0.6)	0.67	0.54	0.56	0.59	0.50	0.60	85.1	77.8	88.3

### 3.3 Study 2: comparing model performance with varying sample sizes

In this study, we evaluated the model's performance across a range of sample sizes, with *N*= 250, 500, and 1,000. For each dataset of a given sample size, we evaluated the model's performance under varying censoring rates of 20%, 40%, 60%, and 85%. Results for 10,000 observations from study 1 were added as a comparison.

**Figure 3:**
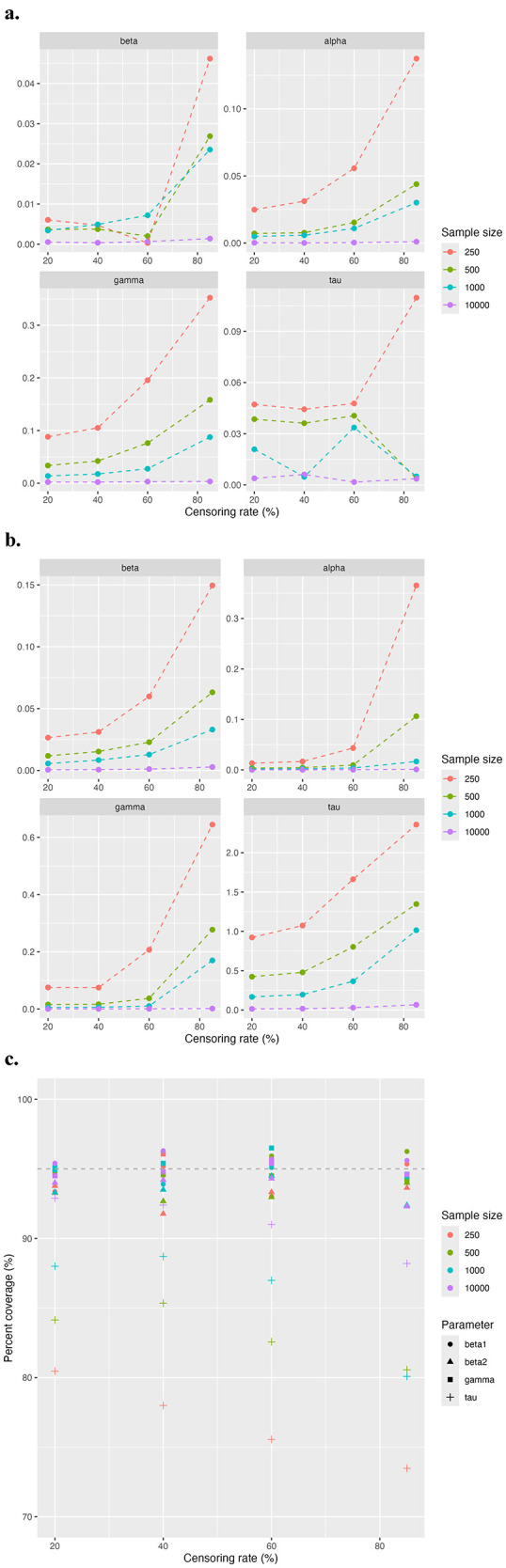
Simulation results for study 2 comparing model performance across different sample sizes. The evaluation metric includes **(a)** absolute bias, **(b)** MSE, and **(c)** coverage rate of 95% CI.

Figure 3 demonstrates the overall model performance across all censoring rates for different sample sizes, focusing on absolute bias (Figure 3a), MSE (Figure 3b), and 95% CI coverage rate (Figure 3c). As expected, the overall model performance decreases as the sample size diminishes and the censoring rate increases. The absolute bias and MSE show similar trends. For larger sample sizes, such as *n* = 500 and *n* = 1, 000, the model maintains a good estimation efficiency even under high censoring rates of 60% to 85%. However, for the smaller sample size at *n* = 100, acceptable estimation efficiency is achieved at moderate censoring rates of 40% to 60%. As in the first study, the 95% CI coverage rates for β, α, and γ remain close to 95% across all conditions. While for τ, the coverage rate declines with increasing censoring rates and smaller sample sizes.

## 4 Application to an occupational epidemiological study dataset

### 4.1 Silicosis dataset

The real-world application we present in this paper illustrates the application of the threshold Cox model to identify the change point in the exposure-response relationship between crystalline silica exposure and silicosis diagnosis. We utilized the same dataset previously analyzed by Birk et al. (12) and (1). The analysis cohort consists of over 17,000 porcelain production workers from over 100 porcelain manufacturing plants in the western states of Germany, who participated in an initial medical screening for silicosis between January 1, 1985, and December 31, 1987. The follow-up period was extended through the end of 2020, or until the worker was diagnosed with silicosis or dropped out of the study, whichever occurred first. During the follow-up, participants were required to receive a chest radiograph (x-ray) every 3 years to monitor for signs of silicosis. In addition, medical records prior to 1975 were retrieved by the Berufsgenossenschaft der keramischen und Glas-Industrie (BGGK), which provides insurance coverage and safety services to workers in the ceramic and glass industries. A rigorous two-stage radiographic review process was used to diagnose silicosis, and in our analysis, a diagnosis was considered positive if either of the two readings indicated silicosis (13). The final cohort contains a total of 17,592 observations, with 156 confirmed cases of silicosis.

Although all cohort members were employed in porcelain manufacturing, only a subset of those who worked directly on the processing line were substantially exposed to crystalline silica and therefore the majority were not at increased risk of the outcome. To account for this and focus the analysis on relevant exposures, we generated a sampled dataset from the total cohort to perform the Cox threshold analysis. The sampled dataset was constructed using a nested case-control sampling approach, with each case randomly matched to 4 controls from the same production departments. The final analysis dataset consists of 748 observations, including 156 cases; however, only 1 control could be matched to 3 cases due to limited availability of appropriate matches.

**Figure 4:**
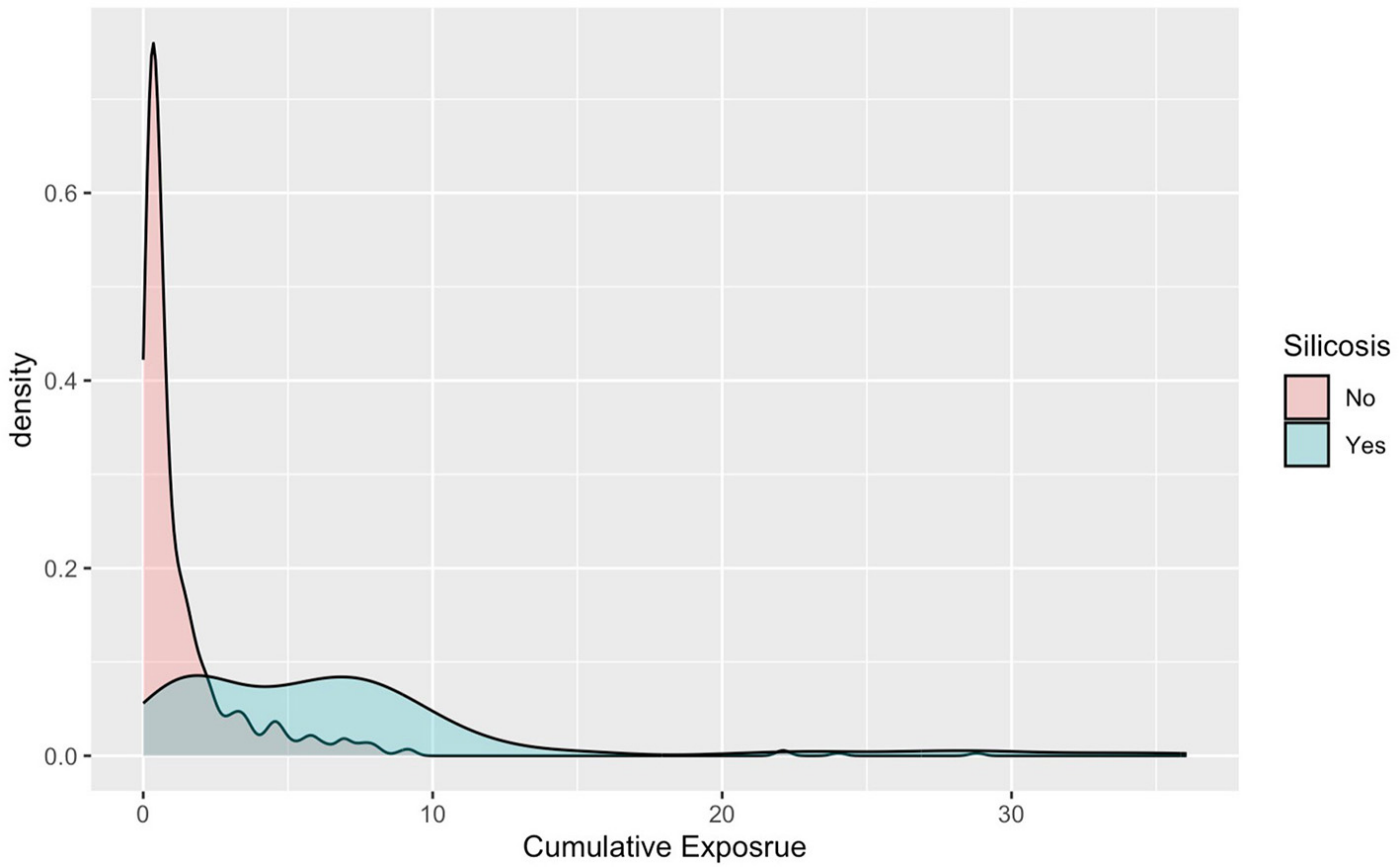
Density plot showing the distribution of cumulative exposure related to silicosis. Two curves are presented: red for those without silicosis and blue for those with silicosis. Distribution of cumulative crystalline silica exposure among silicosis cases and non-cases.

Figure 4 shows the distribution of cumulative crystalline silica exposure between individuals diagnosed with silicosis and those without silicosis. The clear separation between the two distributions indicates a distinct difference in exposure levels, suggesting that higher cumulative exposure is potentially associated with the onset of silicosis, even among those with comparable exposure opportunity.

### 4.2 Exposure threshold modeling

We applied the threshold Cox model containing only exposure to analyze the data. The model takes the formula $\lambda \left(t\right)=\lambda_{0}\left(t\right)\exp\left\{\alpha Z+\gamma {\left(Z-\tau \right)}^{+}\right\}$, where *Z* represents the cumulative crystalline silica exposure. We implemented the two-step grid search method and the one-step maxLik() approach to estimate the model parameters. The parameter estimates are summarized in Table 2. For both methods, the threshold parameter τ is estimated to be 4.04 mg/m^3^. However, the standard error (SE) for τ differs significantly between the methods, with the grid search method yielding a much larger SE of 2.175 based on 1,000 bootstrap resampling, resulting in a wider 95% CI. In contrast, the maxLik() method produces a much smaller SE of 0.474 and a narrower 95% CI. Bootstrap estimates the empirical distribution of the τ by resampling that captures additional sources of variability, and provides a data-driven assessment of uncertainty. The MLE-based variance estimation, on the other hand, relies on asymptotic theory and assumes the model is correctly specified. In our analysis, as the sample size is large enough and we are fairly confident on the model assumptions, the variance estimated with maxLik() is preferred. The estimates for the exposure parameter α and the post-threshold slope γ are consistent across methods, with $\hat{\alpha }\approx$ 0.696 and $\hat{\gamma }\approx$ −0.644. Both methods show similar SEs and CIs for these parameters, indicating stable estimates regardless of the method used.

**Table 2:** Parameter estimation for silicosis analysis.

**Method**	**Parameter**	**Coef**	**SE**	**95% CI**
Grid search	τ	4.040	2.175^*^	(1.223, 9.025)^*^
	α	0.696	0.065	(0.569, 0.823)
	γ	−0.643	0.071	(−0.782, −0.504)
maxLik()	τ	4.038	0.474	(3.109, 4.967)
	α	0.697	0.106	(0.489, 0.905)
	γ	−0.644	0.107	(−0.854, −0.434)

* indicated results from 1,000 non-parametric bootstrap resampling.

We also applied the threshold Cox model using average exposure intensity. Unlike the original analysis, we redefined average RCS exposure intensity based on estimated exposures sustained in the first two and first 5 years of employment. These time-periods were selected because analysis of average RCS exposure estimates by year of employment indicated that most workers demonstrated dramatic reductions in exposure after the first several years of employment. Averaging intensities over their entire employment duration resulted in greatly diluted annual average intensity (note that this phenomenon has minimal effect on cumulative exposure).

Table 3 summarizes parameter estimates for cumulative exposure and for annual exposure intensity at 2 years and 5 years, respectively. The models based on annual exposure intensity show consistent threshold values across the 2-year and 5-year periods (0.264 mg/m^3^ and 0.324 mg/m^3^, respectively), suggesting that disease risk increases once average yearly exposure exceeds these levels. The threshold value identified using cumulative exposure for 2-year and 5-year periods is 0.527 mg/m^3^-yrs, and 1.623 mg/m^3^-yrs. Dividing each cumulative exposure by its corresponding time period results in exactly 0.264 mg/m^3^-yrs and 0.324 mg/m^3^-yrs, demonstrating consistency in the model estimates across different exposure metrics.

**Table 3:** Estimated threshold value and other model parameters via maxLik() for cumulative exposure and annual exposure intensity for the silicosis dataset.

**Exposure type**	**Time period**	**Parameter**	**Coef**	**SE**	**95% CI**
(a) Cumulative exposure	2 yr	τ	0.527	0.058	(0.413, 0.641)
		α	5.052	0.645	(3.788, 6.316)
		γ	−4.338	0.676	(−5.663, −3.013)
	5 yr	τ	1.623	0.154	(1.321, 1.925)
		α	1.757	0.187	(1.391, 2.123)
		γ	−1.517	0.201	(−1.911, −1.123)
(b) Annual exposure intensity	2 yr	τ	0.264	0.029	(0.208, 0.320)
		α	10.086	1.279	(7.579, 12.593)
		γ	−8.666	1.343	(−11.299, −6.033)
	5 yr	τ	0.324	0.031	(0.263, 0.385)
		α	8.793	0.933	(6.964, 10.622)
		γ	−7.594	1.003	(−9.560, −5.628)

## 5 Discussion

In this paper, we described a new threshold Cox model that can estimate a change point in the exposure-response relationship between occupational exposure and a relatively rare outcome of interest. The change point, or threshold, describes the point on the exposure axis where the hazard ratio for silicosis significantly departs from the “background” risk. We proposed two estimation approaches: the two-step grid search method and the one-step MLE using R function maxLik(). While the grid search method is straightforward, obtaining the variance estimate for the threshold parameter τ through bootstrap resampling is time-consuming and computationally intense. On the other hand, the one-step MLE approach can provide point estimates and confidence intervals for all model parameters simultaneously. Under monotone likelihood, the MLE can be biased, and Firth's penalized MLE is incorporated to correct such bias.

We conducted two simulation studies to assess the robustness of the model across different sample sizes with varying censoring rates. In Study 1, we evaluated the performance of the model across different censoring rates at a large sample size *N* = 10,000, focusing on absolute bias, MSE, and 95% CI coverage for key parameters. The model performed well under moderate censoring rates, maintaining low bias and reasonable coverage probabilities for most parameters. As expected, extreme censoring approaching or exceeding 98% resulted in substantial increases in both bias and MSE. While the penalized MLE reduced bias and improved estimation efficiency, it led to a drop in 95% CI coverage probability for τ. This suggests a trade-off between reducing bias and maintaining coverage, which was not widely addressed in previous publications on penalized MLE applications. Study 2 examined the impact of varying sample sizes on the model's performance under different censoring rates. As anticipated, model performance for smaller datasets typically were inferior compared to larger datasets with the same censoring rate. The results from this study highlight several important considerations for applying the threshold Cox model in practice. When the sample size is around 250, the model performs well with a censoring rate below 40-60%. As the sample size increases to around 500 to 1,000, the model maintains strong performance even under moderate to high censoring rates. Practitioners should be mindful of these limitations and consider both sample size and censoring rate when applying the model in real-world scenarios.

In addition to the simulation studies, we also demonstrated the application of the threshold Cox model using a real-world example. In the silicosis dataset, we successfully estimated a threshold of 4.04 mg/m^3^-yrs 95% CI is (3.109, 4.967) for cumulative exposure of crystalline silica, with consistent results across the grid search approach and the maxLik() estimation. Threshold values estimations based on cumulative exposure and annual exposure intensity for the same time period are consistent, indicating the model's ability to reliably estimate the threshold across different exposure metrics. Such results provide a different perspective for evaluating exposure-response relationships where the risk is not best described using a strictly linear function. The parameter estimates from both datasets provide evidence of a change point in the risk associated with increasing exposure, with exposure-response relationships possibly plateauing, declining or increasing linearly beyond the initial threshold. The plateau pattern is observed from the silicosis analysis, which is suggested by the positive estimated α values and the negative estimated γ values, which are of similar magnitude to α. This trend is different from the findings from the Cox model with categorized exposure levels as demonstrated in the analysis by Birk et al. (1). We verified the trend estimated from the threshold Cox model through additional analyses with modeling exposure quintiles and spline models (results not shown). These alternative methods consistently supported the slower increased risk beyond the estimated threshold.

Several limitations emerged during the analysis that may shed light on future research opportunities. The current model only accounts for right-censored observations; however, in occupational studies, it is common to encounter left-truncated datasets. Left truncation occurs when individuals experience the event of interest before the observation period begins, thus excluding them from the final sample and potentially introducing survival bias. For example, in the silicosis analysis, only workers who survived until 1985 are included in the dataset, while those who died of silicosis prior to 1985 are absent from the analytic cohort. Extending the model to accommodate left-truncated data would allow for an estimation procedure more closely aligned with the true underlying population.

The main challenge encountered was the convergence issues in models with extreme censoring, as observed in the simulation study with a 98% censoring rate. This likely helps explain why an earlier attempt to estimate exposure thresholds was unable to derive an estimate for cumulative RCS exposure (14). Although we applied a penalized maximum likelihood approach to address this, the trade-off was a decreased coverage probability, for τ particularly. Future research should explore alternative methods to handle extreme censoring more effectively. For this study, we generated a subset of the original cohort to maximize our ability to differentiate generally high RCS exposures that increased silicosis risk. Potential additional statistical approaches include refining penalization techniques or employing Bayesian methods, both of which could yield more robust estimates for highly censored data.

In summary, the proposed threshold Cox model extends the traditional Cox model by enabling more accurate estimation of change points in the exposure-response relationship. Simulation studies demonstrated strong model performance under varying censoring rates, and the real-world application illustrated its utility in occupational epidemiology. Future methodological developments could focus on enhancing the model's ability to handle left-truncated datasets and refining estimation techniques to better address challenges posed by extreme censoring.

## Data Availability

The data analyzed in this study is subject to the following licenses/restrictions: the datasets presented in this article are not readily available because they are owned by the German Social Accident Insurance (DGUV) of the BG Administrative Sector (VBG). Requests to access these datasets should be directed to Kenneth A. Mundt, kmundt@umass.edu.
