# Supplementary material for: Using threshold Cox models to estimate change points in exposure-response relationships in an occupational epidemiological study of respirable crystalline silica and silicosis risk
Source: Front Public Health. 2025 Sep 19;13:1628965. doi: 10.3389/fpubh.2025.1628965 (PMC12491331; doi:10.3389/fpubh.2025.1628965)
Supplement: Supplementary file 1 [file Data_Sheet_1.pdf]

Supplementary Material for “Using Threshold Cox Models to Estimate Change Points in Exposure-Response Relationships in an Occupational Epidemiological Study of Respirable Crystalline Silica and Silicosis Risk” by Diezhang Wu, Kenneth A. Mundt and Jing Qian.

1 SUPPLEMENTARY DATA

In this vignette, we demonstrate how to estimate change point and all other parameters via `maxLik()` with a simulated dataset.

```
###########################
##### functions used ######
###########################
# 1. function to simulate a survival dataset
generate_surv_data <- function(N, tau, seed){
  set.seed(seed)
  n <- N  # number of observations
  alpha <- 0.75
  beta <- 0.25
  gamma <- 0.5
  tau.0 <- tau  # value of true change point
  x1 <- rbinom(n, 1, 0.55) # mimic male/female, male=1, female=0
  z1 <- rexp(n, rate=0.5) # simulated exposure value
  z2 <- pmax(z1 - tau.0, 0)

  epsilon <- log(rexp(n)) # error term
  predictor.zz <- alpha*x1 + beta*z1 + gamma*z2

  # generate survival time
  tt <- ((exp(-predictor.zz + epsilon))^(0.5) + 2)^(2) # event time
  cc <- runif(n, 4, 18) # censoring time
  xx <- pmin(tt, cc) # observed time
  delta <- 1 * (tt <= cc) # censoring indicator, = 1 for event, = 0 for
     censored observation

  simu_dat <- as.data.frame(cbind(xx, delta, x1, z1))
  colnames(simu_dat) <- c("time", "event", "x1", "z1")

  return(simu_dat)
```

```
}

# 2. Function to calculate partial log-likelihood
LL <- function(param, data){
  # set up the four model parameters to be estimated
  alpha <- param[1]
  beta <- param[2]
  gamma <- param[3]
  tau <- param[4]
  # Apply bounds for tau
  tau <- max(min(param[4], max(data$z1)), min(data$z1))

  # create risk set and compute corresponding partial log-likelihood values
  event <- data$event
  time <- data$time
  x1 <- data$x1
  z1 <- data$z1
  data$z2 <- pmax(data$z1 - tau, 0)
  z2 <- data$z2
  dat_event <- data[data$event == 1,]
  risk_set <- list()
  risk_set_ll <- rep(0, nrow(dat_event))
  for (i in 1:nrow(dat_event)){
        risk_set[[i]] <- data[data$time >= dat_event$time[i], ]
        risk_set_ll[i] <- log(sum(exp(alpha*risk_set[[i]]$x1 + beta*risk_set
           [[i]]$z1 + gamma*risk_set[[i]]$z2)))
  }

  # sum over risk sets to calculate partial log-likelihood value
  llvalue <- sum(event*(alpha*x1 + beta*z1 + gamma*z2)) - sum(risk_set_ll)

  return(llvalue)
}


# 3. Function to calculate gradient function of the partial log-likelihood
gradient_LL <- function(param, data) {
  # set up the four model parameters to be estimated
  alpha <- param[1]
  beta <- param[2]
  gamma <- param[3]
  tau <- param[4]
  tau <- max(min(param[4], max(data$z1)), min(data$z1))

  # create risk set and calculate gradient for each parameter
```

```
  event <- data$event
  time <- data$time
  data$z2 <- pmax(data$z1 - tau, 0)
  dat_event <- data[data$event == 1,]
  risk_set <- list()
  gradient <- rep(0, length(param)) # gradient is a vector with 4 elements
  for (i in 1:nrow(dat_event)){
        risk_set[[i]] <- data[data$time >= dat_event$time[i], ]
        risk_set[[i]]$risk_set_hazard <- exp(alpha*risk_set[[i]]$x1 + beta*risk_set[[i]]$z1 + gamma*risk_set[[i]]$z2)

        gradient[1] <- gradient[1] + dat_event$x1[i] - sum(risk_set[[i]]$x1*risk_set[[i]]$risk_set_hazard)/sum(risk_set[[i]]$risk_set_hazard) # gradient for alpha

        gradient[2] <- gradient[2] + dat_event$z1[i] - sum(risk_set[[i]]$z1*risk_set[[i]]$risk_set_hazard)/sum(risk_set[[i]]$risk_set_hazard) # gradient for beta

        gradient[3] <- gradient[3] + dat_event$z2[i] - sum(risk_set[[i]]$z2*risk_set[[i]]$risk_set_hazard)/sum(risk_set[[i]]$risk_set_hazard) # gradient for gamma

        gradient[4] <- gradient[4] + (-gamma)*ifelse(dat_event$z1[i] > tau, 1, 0) - sum(-gamma*ifelse(risk_set[[i]]$z1 > tau, 1, 0)*risk_set[[i]]$risk_set_hazard)/sum(risk_set[[i]]$risk_set_hazard) # gradient for tau
  }

  return(gradient)
}

##########################################
#### Example of maxLik() estimation ####
##########################################
## Load libraries
library(maxLik)
library(dplyr)
## Simulate a survival data with 10000 observations and a real change point tau=4
surv_data <- generate_surv_data(N = 10000, tau = 4, seed = 12)
## Estimate the model parameters and change point with maxLik()
maxlik_result <- maxLik(logLik = LL, grad = gradient_LL, start = c(0.7, 0.2, 0.45, 3.5), data = surv_data)
summary(maxlik_result)
```

```
## --------------------------------------------
## Maximum Likelihood estimation
## Newton-Raphson maximisation, 4 iterations
## Return code 8: successive function values within relative tolerance limit (reltol)
## Log-Likelihood: -63355.81
## 4  free parameters
## Estimates:
##      Estimate Std. error t value Pr(> t)
## [1,]  0.75964        0.02369   32.06  <2e-16 ***
## [2,]  0.24469        0.01222   20.02  <2e-16 ***
## [3,]  0.49670        0.02108   23.56  <2e-16 ***
## [4,]  3.86273        0.10453   36.95  <2e-16 ***
## ---
## Signif. codes:  0 '***' 0.001 '**' 0.01 '*' 0.05 '.' 0.1 ' ' 1
## --------------------------------------------
```
